# Mapping Smoking Addiction Using Effective Connectivity Analysis

**DOI:** 10.3389/fnhum.2016.00195

**Published:** 2016-05-04

**Authors:** Rongxiang Tang, Adeel Razi, Karl J. Friston, Yi-Yuan Tang

**Affiliations:** ^1^Department of Psychology, Washington University in St. LouisSt. Louis, MO, USA; ^2^The Wellcome Trust Centre for Neuroimaging, University College LondonLondon, UK; ^3^Department of Electronic Engineering, NED University of Engineering and TechnologyKarachi, Pakistan; ^4^Department of Psychological Sciences, Texas Tech UniversityLubbock, TX, USA

**Keywords:** dynamic causal modeling (DCM), smoking addiction, medial prefrontal cortex (mPFC), posterior cingulate cortex (PCC), effective connectivity analysis

## Abstract

Prefrontal and parietal cortex, including the default mode network (DMN; medial prefrontal cortex (mPFC), and posterior cingulate cortex, PCC), have been implicated in addiction. Nonetheless, it remains unclear which brain regions play a crucial role in smoking addiction and the relationship among these regions. Since functional connectivity only measures correlations, addiction-related changes in effective connectivity (directed information flow) among these distributed brain regions remain largely unknown. Here we applied spectral dynamic causal modeling (spDCM) to resting state fMRI to characterize changes in effective connectivity among core regions in smoking addiction. Compared to nonsmokers, smokers had reduced effective connectivity from PCC to mPFC and from RIPL to mPFC, a higher self-inhibition within PCC and a reduction in the amplitude of endogenous neuronal fluctuations driving the mPFC. These results indicate that spDCM can differentiate the functional architectures between the two groups, and may provide insight into the brain mechanisms underlying smoking addiction. Our results also suggest that future brain-based prevention and intervention in addiction should consider the amelioration of mPFC-PCC-IPL circuits.

## Introduction

Tobacco use is a leading preventable cause of death. However, over 90% of smokers try repeatedly to quit but often fail (Centers for Disease Control and Prevention, 2006; Hajek et al., 2009). Nicotine, a component of tobacco, is the primary reason that tobacco is addictive. From the perspective of public health, there is an urgent need to address these serious issues in smoking addiction. Prefrontal and parietal cortex, including the default mode network (DMN); medial prefrontal cortex (mPFC), and posterior cingulate cortex (PCC), anterior cingulate cortex and limbic areas have been shown to involve in addiction (Baler and Volkow, 2006; Hong et al., 2009; Jarraya et al., 2010; Goldstein and Volkow, 2011; Tang et al., 2013, 2015a; Leech and Sharp, 2014; Liang et al., 2015; Weiland et al., 2015). Nonetheless, it remains unclear which brain regions play a crucial role in smoking addiction and the relationship among these regions.

Functional connectivity has been used to examine the intrinsic brain networks related to smoking addiction (Hu et al., 2015; Weiland et al., 2015). However, functional connectivity does not support inferences about causal or directed connectivity. Therefore, any changes in information flow among the brain areas implicated in smoking remain unclear. This limitation calls for a new solution that can characterize causal interactions. Dynamic causal modeling (DCM) has the capacity to identify the causal (directed) connections among distributed brain areas—known as effective connectivity. Spectral DCM (Friston et al., 2014a,b) is especially suited for resting state Functional magnetic resonance imaging or functional MRI (fMRI) that can be summarized with cross spectra. In other words, spectral dynamic causal modeling (spDCM) estimates the effective connectivity among coupled brain regions, which subtends the observed functional connectivity in the frequency domain. Crucially, spectral DCM not only furnishes an efficient estimation of DCM parameters but also enables the detection of group differences in effective connectivity, the amplitude of endogenous neuronal fluctuations or both. It has been shown that spDCM is not only more accurate but also more sensitive to group differences, when compared to stochastic DCM (Razi et al., 2015).

In the current study, we focused on the differences of effective connectivity using spectral DCM at rest between the groups of smokers and non-smokers. We recruited 30 adults (15 smokers and 15 nonsmokers) and applied spectral DCM to resting state fMRI data to quantify the effective connectivity among core regions implicated in smoking addiction. We hypothesized that—in comparison with nonsmokers—smokers would show a disrupted equilibrium between intrinsic (within region) excitatory and inhibitory connectivity—and abnormalities in extrinsic (between region) connectivity, associated with mPFC-PCC-IPL circuits.

The default mode and its connectivity has provided a useful focus for many studies of dysconnectivity in normal subjects and psychopathology. In this work, we characterized coupling within the nodes of the default mode to establish its predictive validity in relation to addictive traits. Our motivation for examining the DMN was two-fold. First, many of the constituent nodes in the DMN have been implicated in addiction. Second, the resting state paradigm is simple and reproducible. In other words, establishing the predictive validity of resting state effective connectivity—as a biomarker in addiction research—may have useful implications for neurogenetic and clinical studies. However, the shortcomings of resting state fMRI studies should be acknowledged. This follows from the fact that endogenous fluctuations in the resting state do not necessarily engage those areas implicated in the functional anatomy of interest. In other words, by restricting our focus to an intrinsic brain network, we cannot guarantee that key connections responsible for executive control and decision making are estimated efficiently. Put simply, studying resting state functional connectivity is a little like “looking for keys under the lamppost”. With this qualification in mind, we now turn to the evidence that many of DMN nodes have a direct relevance for impulsive behavior, attentional deployment and addiction.

Here we have focused on cardinal regions that constitute key nodes of DMN, where these regions have been previously implicated in addiction. The default mode has been implicated in introspective cognition and perspective taking (Amft et al., 2015; Konishi et al., 2015). Crucially, the integration between the salience system and default mode may play a key role in addiction and the moderation of impulsive behavior—as has been demonstrated in the context of cocaine addiction (Liang et al., 2015). This integration between internal and externally directed processing is further substantiated by recently reported reductions in executive and default network functional connectivity in smokers (Weiland et al., 2015). Furthermore, the addictive behavior may be related to a suspension of—or aberrant—reality testing, recent evidence points to the key role of the default mode (in particular, the medial prefrontal cortex) in reality monitoring, relative to source monitoring (Metzak et al., 2015). Clearly, this subset of regions does not provide an exhaustive characterization of the distributed networks implicated in addiction and behavioral control. A pragmatic reason we focused on nodes within the default mode is that these are the regions that were engaged during our resting state study. This is an important aspect of effective connectivity in the following sense: effective connectivity is inherently context sensitive. In other words, it can change with experimental condition, cognitive set and many other factors. This means that the effective connectivity assessed in the current report is specific to the resting state—and is only meaningfully evaluated among regions that show (endogenous) fluctuations in coupled neuronal activity. This is why we focused on components of the default mode that are implicated in addiction. The alternative approach would be to selectively engage regions known to be involved in addiction (and smoking) using a task-based paradigm that selectively activates key regions and implicitly engages effective connectivity among these regions. We will pursue this approach in subsequent work. Comparing the results of effective connectivity analyses between resting state and tasks based studies will be an interesting endeavor and will, hopefully, establish the construct validity of one in terms of the other.

## Materials and Methods

### Subjects

Healthy college students, including smokers and nonsmokers, were recruited through campus advertisements. Among those who responded, we randomly assigned 15 cigarette smokers to one group and 15 nonsmokers to another group (mean ages, 21.30 ± 2.43 years, 20 men), there is no significant difference in age, gender and education between two randomized groups (all *p* > 0.05). We used the widely used Fagerström Test for Nicotine Dependence and carbon monoxide monitor to measure smoking addiction and severity (Heatherton et al., 1991; Deveci et al., 2004). The smokers used tobacco without other drugs, with an average of 10 cigarettes per day. The experiment was approved by the local institutional review board at Texas Tech University, and informed consent was obtained from each participant.

### Neuroimaging

All data were collected using a 3-Telsa Siemens Skyra MRI scanner at the Texas Tech University. A 3D T1-weighted anatomical images were acquired using the MPRAGE sequence (Repetition time (TR) = 1, 780 ms; Echo time (TE) = 2.36 ms; slice thickness = 1.0 mm). A 6-min resting-state functional scan (T2*-weighted images) was obtained for each participant using a gradient echo planar sequence (TR = 2000 ms; TE = 27 ms; flip angle = 80°; field of view (FOV) = 256 mm × 256 mm; matrix size = 64 × 64; slice thickness = 4 mm; Axial direction, 36 slices). Participants looked at a crosshair shown on a screen and were instructed not think of anything in particular. Head movement was minimized with individually custom-made foam padding (Fox and Raichle, 2007). We obtained 28 usable imaging time-series with 14 smokers and 14 nonsmokers for DCM analysis.

Functional data were processed using the Data processing assistant for resting-state fMRI^1^, which is based on SPM^2^ and resting-state fMRI data analysis toolkit (Song et al., 2011). For each participant, the subsequent standard procedures included slice timing, motion correction, regression of WM/CSF signals, and spatial normalization of images into the Montreal Neurological Institute template with a resampling voxel size of 3 × 3 × 3 mm. Finally, a Gaussian filter of 5 mm full-width at half-maximum (FWHM) was applied to the dataset for spatial smoothing (Tang et al., 2013). Our main analysis used spectral DCM as implemented in SPM12.

### ROI Selection

**Figure 1:**
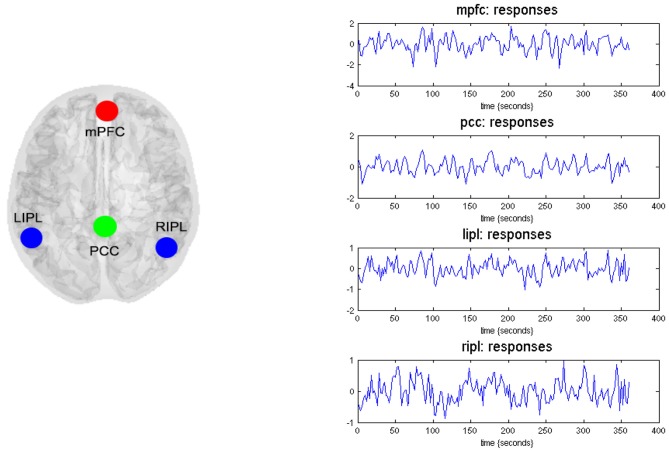
**Four nodes of dynamic causal modeling (DCM) model.** The left panel shows the medial frontal cortex (MFC), the posterior cingulate cortex (PCC), the left inferior parietal lobule (LIPL), and the right inferior parietal lobule (RIPL). The time series (right-hand panels) from four regions are the principal eigenvariates of regions identified using seed connectivity analyses for a typical subject. These time series we used to invert the spectral DCM with the (fully-connected) architecture.

Based on previous literature in addiction fields (Goldstein and Volkow, 2011; Volkow et al., 2012; Tang et al., 2015a), we identified four ROIs including the mPFC, PCC, left and right inferior parietal lobule (L-IPL and R-IPL) as key nodes for effective connectivity analysis. These analyses assess the causal interactions across these regions, as well as the amplitude of endogenous neuronal fluctuations within each region (Di and Biswal, 2014; Razi et al., 2015). To identify nodes of the DMN, the resting state was modeled using a GLM containing a discrete cosine basis set with frequencies ranging from 0.0078 to 0.1 Hz (Fransson, 2005; Kahan et al., 2014), in addition to the nuisance regressors that include the six head motion parameters, CSF and WM regressors. Six head motion parameters were also added into the model to remove potential confounding variances caused by head motion. Data were high-pass filtered to remove any slow frequency drifts (< 0.0078 Hz) in the normal manner. An F-contrast was specified across the discrete cosine transforms (DCT), producing an SPM that identified regions exhibiting blood oxygen level-dependent (BOLD) fluctuations within the frequency band. Our DMN graph comprised of four nodes; the PCC, the LIPL and RIPL), and the mPFC. The PCC node was identified using this GLM: the principal eigenvariate of a (8 mm radius) sphere was computed (adjusted for aforementioned confounds: six head motion parameters and CSF/WM regressors), centered on the peak voxel of the aforementioned F-contrast. The ensuing region of interest was masked by a (8 mm radius) sphere centered on previously reported MNI coordinates for the PCC [0, −52, 26; Di and Biswal, 2014; Razi et al., 2015]. The remaining DMN nodes were identified using a standard seed-based functional connectivity analysis, using the PCC as the reference time series in an independent GLM containing the same confounds. A *t*-contrast on the PCC time series was specified, and the resulting SPM was masked by spheres centered on previously reported coordinates for the RIPC [48, −69, 35], LIPC [−50, −63, 32], and mPFC [3, 54, −2; Di and Biswal, 2014; Razi et al., 2015]. The principal eigenvariate from a (8 mm radius) sphere centered on the peak *t*-value from each region was computed for each region and corrected for confounds. Figure 1 (left panel) shows the 4 nodes of the connectivity model or subgraph. The time series extracted from each of the four regions—for typical subject—are shown in Figure 1 (right panel).

### Dynamic Causal Modeling

We used spectral DCM to analyze the resting state fMRI data. A standard DCM analysis involves a specification of plausible models, which are then allows the model parameters (and subsequent group differences) to be estimated following Bayesian model selection (Friston et al., 2014a; Razi et al., 2015). The first step is to specify a model space. Because there is no previous literature on information transfer within DMN in addiction, we adopted and exploratory approach, starting with a fully connected model. This means that all four ROIs were connected to each other hence there were 16 connectivity parameters (including the recurrent self-connections). It is important to note that the spectral DCM also furnishes parameters that characterize the form of endogenous neuronal fluctuations. These additional parameters model the amplitude and exponent of the neural fluctuations—modeled as power law—for each ROI in the model. Hence, there were 16 connectivity and eight neuronal parameters in our model. Having specified the model, the next step is to estimate or invert the DCM. Model inversion is based on standard variational Bayes procedures (variational Laplace). This approximate Bayesian inference method uses Free Energy as a proxy for (log) model evidence, while optimizing the posterior probabilities (under Laplace approximation) over the model parameters (Friston et al., 2014b).

### Bayesian Model Reduction

In the absence of a particular hypothesis or model space we used the fully connected model for an exploratory analysis of all possible reduced models, without one or more connections: after the full DCM for each participant was inverted, we employed a network discovery procedure using Bayesian model reduction (BMR) (Friston and Penny, 2011) to find the best model that explains the data. This procedure tests every possible model nested within the fully connected model. The model with the highest posterior probability is chosen as the winning model during this procedure. This BMR procedure is an efficient way to score a large model space without having to invert every reduced model. This procedure is based on an approximation, using Savage Dickey density ratio, which allows the computation of the log-evidence of any reduced model, nested within the full model, from the conditional density over the parameters of the full model.

### Inference

Once the winning models for each population are established we can use the parameter estimates from these models to make inference about any group differences. In this work we used Bayesian parameter averaging (BPA; Razi et al., 2015) to quantify group differences in effective connectivity—for each parameter separately: i.e., ignoring posterior correlations (these correlations were subsequently accommodated in a classical multivariate analysis—please see below). This average was calculated for smokers and non-smokers separately. Finally, to test for group differences we used a classical multivariate test—canonical variate analysis (CVA)—to identify significant differences in (mixtures of) model parameters. This multivariate test is inclusive in a sense that it considers all the connections collectively alleviating any need for corrections for multiple corrections.

## Results

### Bayesian Model Reduction

**Figure 2:**
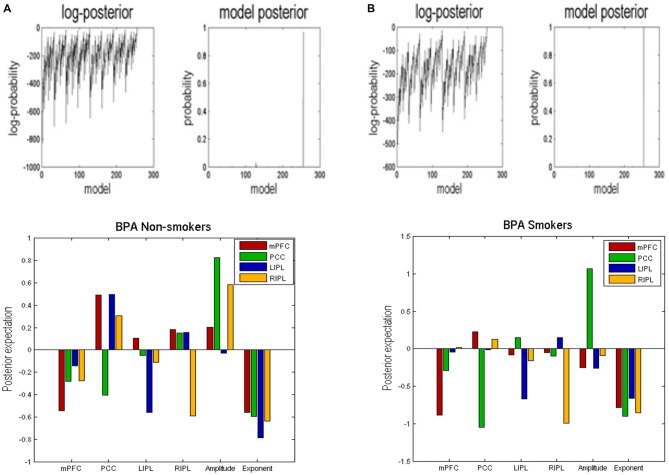
**Bayesian model reduction (BMR).** This figure shows the results of applying BMR procedure. Here column **(A)** is for non-smokers while column **(B)** is for smokers. The upper two panels of column **(A)** (resp. column **B**) show the log-posterior and posterior probability for every model nested within the full model for non-smokers (resp. smokers). The lower panel of column **(A)** (resp. column **B**) shows the Bayesian parametric average (BPA) for the non-smokers (resp. smokers). The horizontal axis show the source region for the 16 connectivity parameters whereas the colors refer to the target regions. We also plotted the eight neuronal parameters showing the amplitude and exponents of the neural fluctuations.

BMR compared the evidence of all reduced models for each group. The results are shown in Figure 2 where left column A is for non-smokers and right column B is for smokers. In both groups, the procedure selected the fully connected model as the best model with a posterior probability of almost 1. The fully connected model had 24 parameters describing the extrinsic connections between nodes, the intrinsic (self-connections) within nodes and neuronal parameters describing the neuronal fluctuations within each node (note that BMR only optimses the connectivity parameter and not neuronal fluctuation parameters). In Figure 2, the profiles of model evidences are shown with the posterior probability for each model. In both groups, the full model had a probability of almost 1 and a log-probability of almost 0. The lower panel of column A (resp. B) shows the Bayesian parametric average for the non-smokers (resp. smokers) of the optimized (full) model. On the horizontal scale, we have the 16 connectivity parameters (which were optimized) and eight neuronal parameters reflecting the amplitude and power law exponent of the neuronal oscillations. The horizontal axis indicates the source regions for the 16 connectivity parameters, while the color indicates the target region. Extrinsic connections have units of hertz (c.f., rate constants), while intrinsic connections and the neuronal estimates are log scaling parameters; in other words, a value of 0.1 corresponds roughly to a 10% increase.

### Bayesian Parametric Averaging

**Figure 3:**
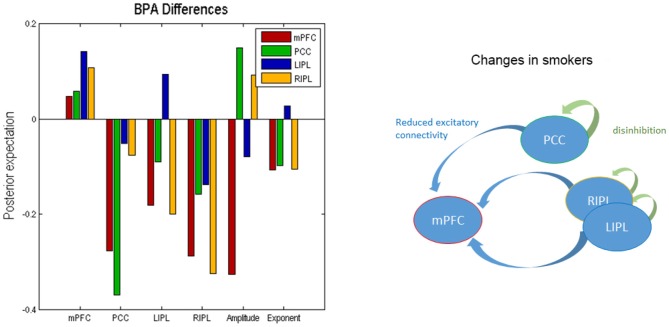
**Bayesian parametric averaging.** This figure shows the Bayesian parametric average (BPA) differences of the two groups. The left panel shows the difference by subtracting the BPA of non-smokers from the smokers. This panel is in the same format as the lower panels of Figure 2. The right panel shows the overall profile of extrinsic and intrinsic differences schematically in the smokers.

We used BPA to summarize the group differences between the smokers and non-smokers. Please note that we used BPA to quantify group differences in effective connectivity– for each parameter separately: i.e., ignoring posterior correlations (these correlations were accommodated in BPA shown in Figure 2 and were also subsequently accommodated in the classical multivariate test). This average was calculated for smokers and non-smokers separately. In Figure 3, we show the difference by subtracting the BPA of non-smokers from the smokers. This means that the positive values on this plot reflects that the connectivity in smokers is greater than non-smokers and* vice versa*. It is in the same format as the lower panels on Figure 3.

In terms of the four self-connections, we see that inhibitory self-connection of PCC showed the largest difference. Since the self-connections in DCM are always inhibitory, this means the responses of the PCC in smokers are disinhibited when compared to non-smokers (by about 30%). We further identified two extrinsic connections—both involving mPFC—that show large differences in connectivity. One is from PCC to mPFC and the other is from RIPL to mPFC. The connection from PCC to mPFC is excitatory for both smokers and non-smokers (see Figure 2) and suggests that smokers have reduced connectivity for these connections as compared to non-smokers. The connection from RIPL to mPFC is inhibitory for smokers and excitatory for non-smokers (see Figure 2) and again shows reduction in connectivity for smokers as compared to controls. The overall profile of extrinsic and intrinsic differences is shown schematically in the right panel of Figure 3. This suggests a functional disconnection of the medial prefrontal cortex from a parietal nodes, which themselves become disinhibited.

In terms of the neuronal parameters, Figure 3 shows that mPFC has the largest (negative) difference. It should to be noted that smokers had negative and non-smokers had positive amplitude scaling for the driving neuronal fluctuations (not shown in Figure 3 as we only show the difference). This means that smokers have reduced neuronal fluctuations in mPFC, as compared to non-smokers. We further note that mPFC also has the largest difference in terms of the power law exponent of the neural fluctuations, suggesting that mPFC may have faster oscillations in smokers as compared to the non-smokers. In other words, the endogenous fluctuations became more slowly as frequency increases. A preponderance of higher frequencies usually indicates more excitable intrinsic neuronal dynamics, which is consistent with a loss of extrinsic entrainment by extrinsic inputs from parietal regions.

### Multivariate Analysis

**Figure 4:**
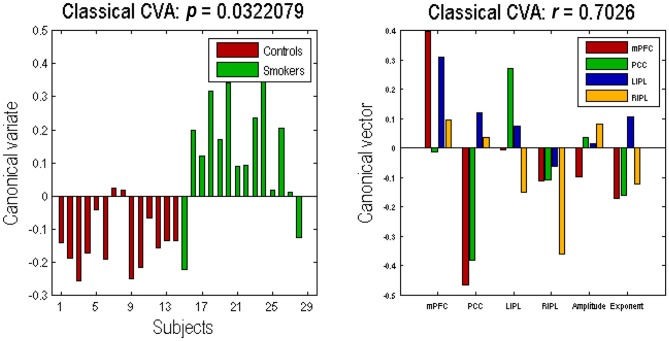
**Canonical variate analysis (CVA).** This figure shows the results of a canonical variates analysis (the multivariate classical inference) using the same summary statistics used in Figure 3. The canonical variate shows reliable group discrimination on the left panel, while the canonical vector shows weights assigned to each parameter by CVA on the right panel (the format of this panel is same as the Figures 2, 3). The effect of group difference was significant with a canonical correlation of *r* = 0.7026; *p* = 0.0322.

The profile of connectivity changes, using BPA, above is purely quantitative. To establish that these differences are significant, in relation to intersubject variability, we used classical tests based on subject specific parameter estimates. Figure 4 shows the results of a classical multivariate test—CVA. We used this analysis to test for any differences over all connections between the groups. The results of CVA include canonical vectors and variates—and their significance. These are plotted on the left and the right panels respectively. First, we see that this test is significant with a *p*-value of 0.032 and a strong canonical correlation *(r)* of 0.702. Note that because there is only one multivariate test, there is no need to correct for multiple comparisons. The canonical variate (shown on the left panel) expresses the degree to which a pattern of differences—encoded by the canonical vector (shown on the right panel)—is expressed in each subject. The left panel shows that, with the exception of couple of subjects in each group, the corresponding canonical variate can reliably discriminate between the two groups. The right panel shows the pattern of weights assigned by CVA to each parameter. It is pleasing to note a very similar pattern here to the one shown in Figure 3. We see that PCC self-connection is the largest difference, which is agreement with BPA results. Also, the connection from PCC to mPFC is given the largest (negative) weight. As for the amplitude and exponent of the neural fluctuations, we again see very similar pattern: the mPFC has the largest differences in smokers (compared to controls), which is consistent with the BPA differences in Figure 3.

## Discussion

In this work, we focused on the implication of DMN on addiction (Posner et al., 2007; Hong et al., 2009; Tang et al., 2010, 2013, 2015b; Goldstein and Volkow, 2011; Petersen and Posner, 2012; Liang et al., 2015; Weiland et al., 2015), and modeled the effective connectivity underlying low frequency BOLD fluctuations in the resting smoker’s brain network. This analysis disclosed the causal and distributed effects of smoking addiction on four core brain regions and their prefrontal-parietal connections. Our results suggest differences in functional integration among brain DMNs between nonsmokers and smokers.

Compared to nonsmokers, smokers showed a reduced excitatory coupling from PCC to mPFC, a reduced coupling from RIPL to mPFC, and disinhibition of both PCC and RIPL. These results suggest smokers lose equilibrium between excitatory and inhibitory connectivity, especially in the mPFC-PCC-IPL circuits. Overall, the results suggest that the medial prefrontal cortex becomes less sensitive to extrinsic afferents from parietal nodes, which themselves are disinhibited. Our findings are consistent with previous neuroscientific research in smoking addiction; for example, smokers often show reduced brain activity in the prefrontal-parietal networks (Hong et al., 2009; Goldstein and Volkow, 2011; Tang et al., 2013, 2015a; Weiland et al., 2015), and after intervention, the brain activity of these networks increases (Tang et al., 2013, 2015a). mPFC and PCC are key nodes of the human default network (Raichle, 2015) which orchestrates the brain’s ongoing or endogenous activity in the resting-state. Previous research has shown that endogenous activity plays a major role in the human brain in health and neurological and psychiatric disorders (Zhang and Raichle, 2010).

Our study reveals changes in the dynamic interplay among mPFC and PCC—two key brain regions involved in smoking addiction (Jarraya et al., 2010; Goldstein and Volkow, 2011), in which smokers have reduced connectivity from PCC to mPFC as compared to non-smokers. Given that PCC has been shown as the brain connector hubs that link all major structural modules and play an important role in functional integration (Hagmann et al., 2008; Zuo et al., 2012), the dysregulation from PCC to mPFC in smoking addiction may be a crucial biomarker. The addicts such as nicotine, cocaine and methamphetamine users show functional and structural abnormalities in the PFC and IPL (Bustamante et al., 2011; Luijten et al., 2013; Hall et al., 2015). For example, the RIPL is often less activated in cocaine-dependent groups during conditions requiring attention and cognitive control (Barrós-Loscertales et al., 2011; Bustamante et al., 2011). However, these studies did not address the directed and dynamic interactions among the brain regions involved. In the current study, we applied spectral DCM to first show a reduced excitatory coupling from RIPL to mPFC, reflecting the directed connectivity and abnormalities of information flow from one area to another, consistent with previous empirical findings. These results may shed light on the potential biomarkers of diagnosis and the target of effective treatment in smoking addiction. Furthermore, they suggest that future brain based prevention and intervention could consider the amelioration of interactions in mPFC-PCC-IPL circuits. Future work should also explore cortico-subcortical interactions in smoking addiction.

In terms of the functional anatomy suggested by our dynamic causal modeling, a key region appears to be the mPFC. This region was unique in showing a reduction in extrinsic afferents from other areas. This finding is particularly interesting given the role of the mPFC in evaluation, reality monitoring, decision-making and choice behavior (Rushworth et al., 2004; Metzak et al., 2015). For example, it has been proposed that the function of the mPFC “is to learn associations between context, locations, events, and corresponding adaptive responses, particularly emotional responses” (Euston et al., 2012). Furthermore, functional connectivity analyses have suggested “that the value signal in VMPFC might integrate inputs from networks, including the anterior insula and posterior superior temporal cortex that are thought to be involved in social cognition” (Hare et al., 2010). To the extent that addictive behavior may be related to a suspension of—or aberrant—reality testing, recent evidence points to the key role of the default mode (in particular, the mPFC) in reality monitoring, relative to source monitoring (Metzak et al., 2015). The particular profile of effective connectivity changes that characterize addicted smokers are also remarkably similar to the decrease in effective connectivity between parietal and mPFC regions in schizophrenia. In a recent stochastic DCM study of the default mode in first episode schizophrenia, the authors found reduced effective connectivity to the anterior frontal node of the default mode—“reflecting a reduced postsynaptic efficacy of prefrontal afferents” (Bastos-Leite et al., 2015).

Does smoking severity correlate with connectivity? We conducted an analysis but did not find the correlation between smoking severity and effective connectivity, possibly because of the small sample size in the current study. Nonetheless, our data indicate the use of spectral DCM on resting-state fMRI data can differentiate the directed connections between two groups, and provide insight into the brain mechanisms underlying smoking addiction; namely, abnormalities of effective connectivity in the brain. Our findings are in accord with our hypothesis that in—comparison with nonsmokers—smokers show a disrupted equilibrium between excitatory and inhibitory connectivity (mPFC-PCC-IPL circuits). This disrupted functional integration can be summarized as a functional disconnection of the medial prefrontal cortex from posterior parietal nodes.

In summary, many psychiatric (and neurological) conditions such as major depressive disorder and schizophrenia can be understood as functional disconnection syndromes (Menon, 2011; Sylvester et al., 2012): effective connectivity can tell us how brain regions interact with each other in terms of context sensitive changes in directed coupling—and even address the relationship between persistent changes in effective connectivity and relapse.

## Author Contributions

All authors listed, have made substantial, direct and intellectual contribution to the work, and approved it for publication.

## Conflict of Interest Statement

The authors declare that the research was conducted in the absence of any commercial or financial relationships that could be construed as a potential conflict of interest.

## Footnotes

^1^www.restfmri.net

^2^www.fil.ion.ucl.ac.uk/spm
